# Lipophagy: A key regulator in oxidative stress and metabolic disorders

**DOI:** 10.1016/j.gendis.2026.102049

**Published:** 2026-01-22

**Authors:** Qingqing Zhao, Fei Qu, Yi Jin

**Affiliations:** Central Hospital Affiliated to Shandong First Medical University, Jinan, Shandong 250013, China

**Keywords:** Lipid droplet, Lipid homeostasis, Lipophagy, Metabolicdisorders, Oxidative stress

## Abstract

Lipophagy, the selective autophagic degradation of lipid droplets, is essential for regulating cellular lipid levels and energy balance while preventing lipotoxicity. This process integrates with metabolic pathways, particularly in counteracting oxidative stress by clearing excess lipids that generate reactive oxygen species. Dysregulation of lipophagy contributes to metabolic disorders, including obesity and diabetes, where impaired lipid clearance leads to insulin resistance, inflammation, and tissue damage. It also exerts protective effects in cardiovascular health by modulating lipid accumulation in atherosclerosis and influences neurodegenerative diseases by alleviating lipid-induced neuronal stress. This review emphasizes the therapeutic potential of targeting lipophagy, proposing that pathway modulation could offer innovative strategies for treating lipid dysregulation-associated diseases. Advancing knowledge of lipophagy's mechanisms and its interplay with oxidative stress may enable therapies that tackle underlying pathologies beyond symptom management.

## Introduction

Lipophagy, the selective autophagic degradation of lipid droplets (LDs), emerges as a critical regulator in cellular lipid homeostasis and energy balance, particularly in mitigating oxidative stress associated with metabolic disorders. Recent advancements underscore its role in preventing lipotoxicity—cellular dysfunction arising from excess lipid accumulation—by facilitating lipid turnover and integrating with broader metabolic pathways.^1^^,^^2^ This review uniquely synthesizes lipophagy's mechanisms with its implications in oxidative stress across diverse diseases, drawing on recent studies to address gaps in prior disease-specific reviews.

LDs, dynamic organelles central to energy provision, signaling, and membrane trafficking,^3^^,^^4^ respond to nutritional or stress cues through selective degradation via lipophagy.^5^ This process ensures that lipids are mobilized for energy while preventing pathological buildup,^6^ with dysregulation leading to lipid depletion or excess, both contributing to disease pathogenesis.^7^^,^^8^

Beyond metabolic regulation, lipophagy influences conditions like obesity, type 2 diabetes, and metabolic dysfunction-associated steatotic liver disease (MASLD, formerly non-alcoholic fatty liver disease),^9^, ^10^, ^11^, ^12^ where impaired clearance causes lipid overload, insulin resistance, inflammation, and tissue damage.^13^, ^14^, ^15^ In atherosclerosis, it modulates macrophage lipid handling, affecting plaque stability and cardiovascular risk.^16^^,^^17^ Emerging evidence also links lipophagy to neurodegeneration, such as in Alzheimer's disease and Parkinson's disease, where it alleviates lipid-induced oxidative stress and neuronal damage, potentially reducing toxic protein accumulation.^18^^,^^19^

The therapeutic promise of lipophagy modulation is substantial, offering strategies to correct metabolic imbalances at their source. Agents targeting key regulators like AMP-activated protein kinase (AMPK, which activates lipophagy under energy stress) and mechanistic target of rapamycin (mTOR, which inhibits it in nutrient abundance) show efficacy in animal models.^20^^,^^21^ For instance, AMPK agonists enhance lipophagy to reduce oxidative stress in MASLD and diabetic kidney disease.^22^^,^^23^ Hormonal and nutritional signals, including insulin (suppressive) and glucagon (promotive), further modulate lipophagy in response to nutrient availability,^24^^,^^25^ underscoring its adaptability to interventions.

Deepening insights into lipophagy's cellular and systemic roles, especially its interplay with oxidative stress, pave the way for innovative therapies in metabolic and degenerative conditions. Subsequent sections detail its mechanisms, pathological contributions, and emerging modulation strategies. This comprehensive overview not only consolidates molecular details, such as LD recognition by adaptors like p62 and lysosomal fusion via Ras-related protein Rab-7 (Rab7), but also explores regulatory networks involving transcription factor EB (TFEB) and peroxisome proliferator-activated receptors (PPARs) for lysosomal biogenesis and fatty acid oxidation. In MASLD, lipophagy impairment exacerbates steatosis-to-steatohepatitis progression through reactive oxygen species (ROS) accumulation, as seen in reduced autophagic flux.^12^^,^^14^ Similarly, in atherosclerosis, defective lipophagy promotes plaque instability via lipid-induced inflammation.^19^ For obesity and diabetes, pathways like AMPK/UNC-51-like kinase 1 (ULK1) restore flux, improving metabolic outcomes in preclinical studies.^21^^,^^23^ Neurodegenerative links highlight lipophagy's role in clearing ROS-damaged components, with potential overlaps in Alzheimer's disease via amyloid–beta interactions.^19^

## Molecular mechanisms of lipophagy

Understanding the molecular mechanisms of lipophagy is fundamental to elucidating its role in cellular lipid metabolism and oxidative stress mitigation. This process involves intricate interactions between LDs, autophagic machinery, and regulatory pathways that respond to metabolic cues. The following subsections detail the structure and function of LDs as dynamic organelles, followed by the step-by-step process of lipophagy, highlighting how these elements contribute to preventing lipid-induced oxidative damage in metabolic disorders.

## Lipid droplet structure and function

LDs, once viewed as inert fat repositories, have emerged as dynamic organelles central to cellular lipid metabolism, energy homeostasis, and oxidative stress mitigation.^7^^,^^8^ Structurally, LDs consist of a hydrophobic core primarily containing neutral lipids, such as triglycerides and cholesteryl esters, surrounded by a phospholipid monolayer embedded with specialized proteins that regulate their formation, growth, and interactions with other cellular compartments.^12^ These proteins include perilipins (PLINs), which coat the droplet surface to control access to internal lipids and facilitate responses to metabolic cues.^14^ LDs are ubiquitous across cell types but are most abundant in adipocytes, where they serve as primary energy reserves.^17^ Primarily, LDs store energy efficiently, releasing fatty acids for β-oxidation during fasting or heightened energy demands.^19^ However, their multifunctionality extends to roles in membrane trafficking, protein storage and degradation, and as hubs for signaling molecules, underscoring their importance in adapting to nutrient fluctuations.^21^ By sequestering excess lipids, LDs prevent lipotoxicity and the associated generation of ROS, which can lead to cellular dysfunction in metabolic disorders.^22^

## Process of lipophagy

Lipophagy, a selective form of macroautophagy, targets LDs for degradation, playing a pivotal role in managing lipid stores, maintaining energy homeostasis, and mitigating oxidative stress in response to nutrient availability and cellular demands. This process prevents lipotoxicity by breaking down excess lipids into free fatty acids and glycerol, which can be utilized for energy production or as precursors for new lipids. Understanding lipophagy requires a foundational overview of autophagy, a conserved cellular mechanism for degrading and recycling components via the lysosomal system, essential for quality control, stress adaptation, and homeostasis.^22^^,^^23^

Autophagy encompasses several types: macroautophagy (bulk degradation via autophagosomes), microautophagy (direct lysosomal engulfment), and chaperone-mediated autophagy (selective protein targeting via chaperones like HSC70).^26^ Macroautophagy, the primary form in lipophagy, involves forming a double-membraned autophagosome that engulfs cargo.

In lipophagy, LD sequestration is selective, mediated by adaptor proteins such as p62/sequestosome 1 (SQSTM1), which binds to microtubule-associated protein 1 light chain 3 (LC3) on autophagosomes and LD-associated proteins like PLIN2.^27^^,^^28^ Ubiquitination signals on LDs enhance targeting by autophagy-related (ATG) proteins.^29^ Autophagosomes then fuse with lysosomes to form autolysosomes, facilitated by soluble N-ethylmaleimide-sensitive factor attachment protein receptors (SNAREs) and Rab7.^30^ In the acidic lysosomal environment, hydrolases like lysosomal acid lipase hydrolyze triglycerides and cholesteryl esters into free fatty acids and cholesterol.^6^

This degradation not only fuels β-oxidation but regulates lipid levels, averting ROS accumulation and oxidative damage in conditions like steatosis or cardiomyopathy.^31^ By elucidating these steps, including LD recognition and autolysosomal breakdown, lipophagy emerges as a therapeutic target for lipid dysregulation-linked diseases.^21^

## Regulation of lipophagy

Lipophagy, akin to other autophagic processes, is governed by a sophisticated network of signaling pathways and transcription factors responsive to cellular and environmental shifts. Comprehending these mechanisms is vital for clarifying lipophagy's contributions to health and pathology, particularly in oxidative stress contexts where it prevents lipid-induced ROS accumulation. The AMPK and mechanistic target of rapamycin complex 1 (mTORC1) pathway are pivotal, often counteracting to align cellular energy demands with nutrient supply.^20^ AMPK acts as an energy sensor, activating under low energy (high AMP/ATP ratios) to foster ATP-generating catabolic processes, including lipophagy.^32^ This occurs via phosphorylation of mTORC1 components like Raptor, inhibiting mTORC1, an autophagy suppressor, and directly activating the ULK1 complex, essential for autophagy initiation.^33^ In contrast, mTORC1, thriving in nutrient-rich environments, serves as a primary autophagy inhibitor, including lipophagy.^34^ mTORC1 phosphorylates ULK1 directly, blocking autophagy onset.^35^ The AMPK–mTORC1 interplay is crucial for metabolic adaptation, stimulating lipophagy during nutrient deprivation and suppressing it when nutrients abound, thereby regulating oxidative stress by modulating lipid turnover.^36^

TFEB and PPARs are instrumental in lipophagy regulation. TFEB oversees lysosome biogenesis and autophagy; normally phosphorylated and cytoplasmic, it dephosphorylates during autophagic induction, translocating to the nucleus to activate genes for autophagy and lysosomal function.^37^ This transcriptional surge is critical for amplifying lipophagy machinery.^24^ PPARs, lipid-sensing nuclear receptors, govern lipid metabolism; PPARα notably induces fatty acid breakdown and lipophagy genes in response to fatty acid levels.^38^ PPARα activation boosts autophagy gene expression and LD degradation, forging a direct link between lipid metabolism and autophagic clearance.^39^ The mTORC1–TFEB–Rag–Ragulator complex further illustrates this, where mTORC1 sequesters TFEB cytosolically, while AMPK may promote TFEB independently of mTOR in some contexts.^40^

**Figure 1:**
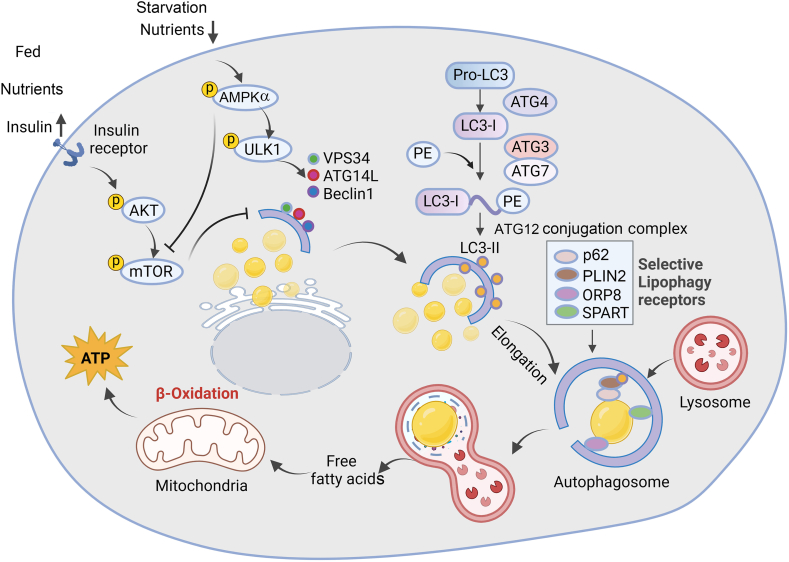
Lipophagy in mammalian cells. Under basal feeding conditions, nutrients, insulin, and growth factors stimulate class I PI3K activity, which in turn activates the mTOR pathway, thereby inhibiting autophagosome formation. Conversely, starvation conditions activate the AMPKα–ULK1 pathway, promoting autophagosome formation. Lipophagy initiation is reliant on various protein complexes, including ULK1, class III PI3K, and ATG proteins, among others. The recruitment of autophagosomal membranes is mediated by adaptor proteins that contain LC3-binding regions, such as p62. P62 is considered a potential lipophagy receptor because it interacts with PLIN2 to bind to LDs and is crucial for the lipophagy process. Furthermore, certain lipophagy receptor proteins, such as ORP8 or SPART, play a crucial role in attracting autophagic membranes containing LC3 to the LDs.

Hormonal and nutritional signals profoundly modulate lipophagy. Insulin typically fosters lipid accrual by activating lipogenic pathways and curbing AMPK, thus diminishing autophagic activity, including lipophagy.^41^ Glucagon, conversely, enhances lipophagy via AMPK activation amid low glucose.^42^ Nutritional cues like amino acid abundance activate mTORC1 to inhibit lipophagy, whereas scarcity inactivates mTORC1, prompting lipophagy.^43^ These networks ensure precise lipophagy control amid diverse metabolic signals, mirroring cellular adaptation to cues. Insights into these pathways reveal how lipophagy disruptions fuel metabolic disorders and pinpoint therapeutic targets. Adjusting these networks could rectify faulty lipophagy-linked issues, offering novel avenues for tackling conditions like obesity, diabetes, and fatty liver disease, especially by alleviating oxidative stress through enhanced lipid clearance (Fig. 1).^44^

## Lipophagy and oxidative stress: A central link in metabolic homeostasis

Lipophagy, the selective autophagic degradation of LDs, serves as a central mechanism in metabolic homeostasis by regulating lipid turnover, energy balance, and cellular adaptation to nutrient fluctuations.^21^ This process not only facilitates the breakdown of excess lipids to prevent lipotoxicity—cellular dysfunction from lipid overload—but also integrates with broader metabolic pathways to mitigate oxidative stress, a hallmark of metabolic disorders.^45^ Oxidative stress arises from an imbalance between ROS production and antioxidant defenses, often exacerbated by lipid accumulation that overwhelms mitochondrial β-oxidation, leading to electron transport chain leakage and superoxide generation.^46^ In healthy states, lipophagy maintains redox equilibrium by mobilizing stored triacylglycerols for energy, reducing LD overload, and preventing ROS-mediated damage to organelles like mitochondria and the endoplasmic reticulum.^47^ Dysregulation of lipophagy disrupts this link, contributing to a vicious cycle where impaired lipid clearance amplifies oxidative stress, inflammation, and metabolic imbalances in conditions such as obesity, type 2 diabetes, MASLD, atherosclerosis, and neurodegenerative diseases.^2^

In metabolic homeostasis, lipophagy acts as a nutrient sensor, responding to energy demands via pathways like AMPK activation during starvation, which promotes LD degradation to fuel β-oxidation and ATP production.^48^ This prevents lipotoxicity-induced ROS from sources such as NADPH oxidases and xanthine oxidase, preserving cellular integrity.^39^ For instance, in hepatocytes, lipophagy coordinates with cytosolic lipolysis (via adipose triglyceride lipase) and very low-density lipoprotein secretion to regulate lipid flux, ensuring systemic energy balance.^43^ Recent studies emphasize lipophagy's role in countering oxidative stress through selective mechanisms: macroautophagy engulfs entire LDs, chaperone-mediated autophagy degrades PLINs to expose lipids, and microautophagy enables piecemeal LD uptake by lysosomes.^49^ These forms integrate to maintain metabolic flexibility, with chaperone-mediated autophagy particularly crucial during prolonged fasting to sustain lipid catabolism without overwhelming mitochondrial capacity.^50^

The connection between lipophagy and oxidative stress is bidirectional and central to metabolic disorders. Impaired lipophagy leads to LD accumulation, promoting lipotoxicity where excess free fatty acids induce mitochondrial dysfunction, ER stress (via unfolded protein response), and ROS amplification.^51^ In MASLD, chronic nutrient excess hyperactivates mTORC1, suppressing TFEB-mediated lysosomal biogenesis and lipophagic flux, resulting in steatosis and ROS-driven progression to steatohepatitis (metabolic dysfunction-associated steatohepatitis).^52^ Oxidative stress further impairs lipophagy by damaging autophagy proteins (*e.g.*, Atg3/Atg7 oxidation) and lysosomes, creating a feedback loop that exacerbates inflammation via NLRP3 inflammasome activation and cytokine release (*e.g.*, IL-1β, IL-18).^53^ In diabetic kidney disease, lipophagy dysfunction in proximal tubular cells causes lipid peroxidation and ROS elevation, disrupting glomerular filtration and promoting fibrosis.^54^ Similarly, in atherosclerosis, defective lipophagy in macrophages leads to foam cell formation and oxidized low-density lipoprotein (LDL)-induced plaque instability.^55^ Neurodegenerative links involve neuronal lipophagy mitigating lipid-induced ROS, with impairments in Alzheimer's and Parkinson's accelerating toxic protein aggregation and neuronal death.^18^

Recent studies underscore this central link, highlighting lipophagy's therapeutic potential in restoring metabolic homeostasis. For example, in MASLD models, lipophagy impairment fosters a lipotoxic environment that heightens oxidative stress through incomplete β-oxidation and ETC leakage, with interventions like AMPK activators breaking this cycle by enhancing LD clearance.^45^ In diabetic kidney disease, natural compounds modulate lipophagy via AMPK/mTOR/ULK1 pathways to reduce renal lipid accumulation and ROS, improving insulin sensitivity and tubular function.^47^ Bibliometric analyses from 2025 reveal “oxidative stress” as a key node connecting autophagy and metabolic clusters in MASLD research, emphasizing multi-target strategies. Emerging findings also show oxysterols (*e.g.*, 25-hydroxycholesterol) inducing oxiapoptophagy—a hybrid of oxidative stress, apoptosis, and autophagy—further tying lipid dysregulation to metabolic imbalance.^46^

Therapeutically, modulating lipophagy addresses the oxidative-metabolic nexus at its root. Agents targeting AMPK (*e.g.*, metformin, resveratrol) enhance lipophagy to alleviate ROS in MASLD and diabetic kidney disease, while mTORC1 inhibitors (*e.g.*, rapamycin) restore flux in steatotic livers.^20^ Natural modulators like berberine and quercetin exhibit dual autophagy–antioxidant effects, up-regulating SOD/CAT/GPX while promoting TFEB nuclear translocation to boost lipophagy and reduce fibrosis.^43^ Challenges include ensuring balanced free fatty acid utilization to avoid mitochondrial overload, with future directions focusing on tissue-specific delivery and biomarkers for autophagic flux.^56^ By enhancing lipophagy, these strategies unlock novel approaches to correct metabolic disruptions, targeting underlying causes rather than symptoms in oxidative stress-driven disorders.^9^

## Lipophagy in metabolic disorders

Lipophagy's dysregulation is implicated in a spectrum of metabolic disorders, where impaired lipid clearance leads to lipotoxicity, oxidative stress, inflammation, and tissue damage. This section examines its roles in key conditions, highlighting mechanistic links to ROS accumulation and potential therapeutic interventions. Beginning with MASLD, we explore how lipophagy dysfunction exacerbates hepatic steatosis, followed by its impacts on atherosclerosis, obesity, diabetes, cancer, neurodegenerative diseases, and renal lipotoxicity. By integrating lipophagy with oxidative stress—a theme underexplored in recent disease-specific reviews—this analysis underscores its central role in metabolic homeostasis and potential as a therapeutic target.

## MASLD

MASLD encompasses a spectrum of liver disorders characterized by excessive fat accumulation in hepatocytes unrelated to alcohol consumption.^57^ Disruption of lipophagy, the selective autophagic breakdown of LDs, is a key factor in MASLD advancement. In healthy livers, lipophagy efficiently regulates lipid turnover in hepatocytes, preventing accumulation and maintaining homeostasis.^21^ However, in MASLD, this process is impaired, leading to hepatic steatosis and potentially severe liver damage like steatohepatitis (metabolic dysfunction-associated steatohepatitis), fibrosis, and cirrhosis.^58^ This dysregulation exacerbates oxidative stress through mitochondrial overload from undegraded lipids, generating ROS via electron transport chain leakage and activating inflammatory pathways like NLRP3 inflammasome.^59^

Disturbances in autophagy contribute to MASLD development. TFEB and transcription factor E3 (TFE3) control lysosome biogenesis and autophagy.^43^ Studies on fenofibrate, a PPARα agonist, in high-fat diet (HFD)-fed mice and Tfeb knockout models showed its effects depend on TFEB.^24^ Fenofibrate reduced obesity and diabetes indicators, liver fat levels, and improved glucose tolerance, insulin sensitivity, and autophagy via TFEB interaction.^24^ It stimulated autophagy and activated TFEB/TFE3, diminishing hepatic fat independently of mTOR.^24^ TFEB inhibition reversed fenofibrate's benefits on autophagy and lipid storage.^24^ Fenofibrate induced lysosomal Ca^2+^ release via mucolipin 1 (MCOLN1), activating calcineurin and calmodulin-dependent protein kinase kinase beta (CaMKKβ)–AMPK–ULK1 pathways, facilitating TFEB/TFE3 dephosphorylation and nuclear translocation.^24^ Calcium chelators or MCOLN1 silencing reversed these effects.^24^ Thus, PPARα activation alleviates hepatic fat via TFEB-mediated lipophagy, critically involving lysosomal calcium signaling.^24^^,^^60^ Harnessing TFEB via PPARα modulation, through drugs like fenofibrate or novel compounds, presents a therapeutic approach for MASLD and metabolic disorders.^61^

Fat buildup in the liver and adipose tissues is central to MASLD progression.^25^ Researchers observed LDL sequestration by autophagic structures and lysosomal breakdown in cells and mice.^6^ p62/SQSTM1 is essential, targeted for enhancing lipophagy.^62^ p62 agonists effectively address liver fat and obesity in models.^54^ The N-degron pathway governs lipophagy: Molecular chaperones like binding immunoglobulin protein (BiP/GRP78) retrotranslocate from the endoplasmic reticulum and are N-terminally arginylated by arginyl-tRNA-protein transferase 1 (ATE1).^9^ N-terminal arginine (Nt-Arg) binds p62's ZZ domain on LDs, triggering p62 polymerization and attracting LC3-positive lipophagosomes for lysosomal degradation.^9^ Liver-specific Ate1 knockout mice on HFD exhibit pronounced MASLD symptoms.^9^ Nt-Arg as a small-molecule p62 agonist enhances lipophagy, reducing obesity and liver fat in wild-type but not p62-deficient mice.^9^ Thus, the N-degron pathway regulates lipophagy and offers pharmacological targets for MASLD.^11^

**Figure 2:**
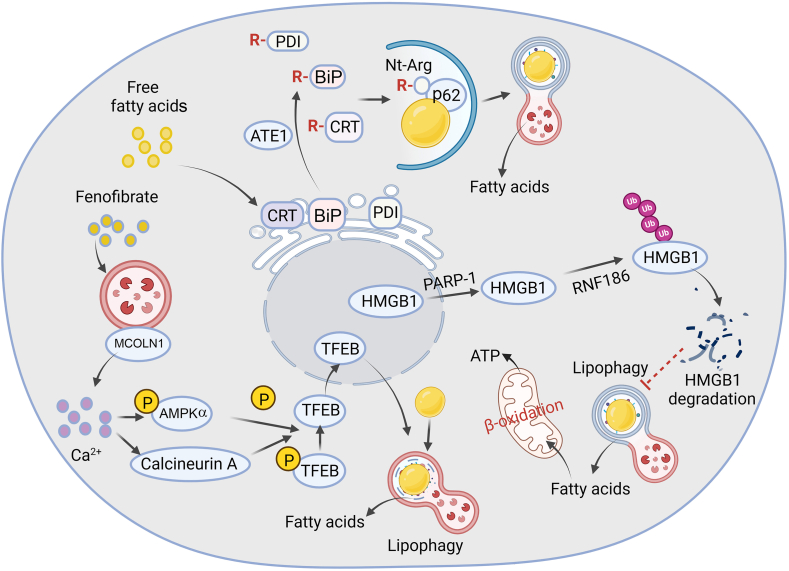
Role of lipophagy in MASLD. i) Fenofibrate initiates lysosomal Ca^2+^ release via MCOLN1, enhancing calcineurin/CaMKKβ–AMPKα–ULK1 pathways for TFEB/TFE3 dephosphorylation/nuclear translocation, promoting lipophagy/lipolysis. ii) N-degron pathway regulates lipophagy: Chaperones (BiP/CRT/PDI) retrotranslocate from the ER, N-arginylated by ATE1. Nt-Arg binds to LD-associated p62's ZZ domain, triggering polymerization and attracting LC3-positive lipophagosomes for degradation. HMGB1 nuclear-to-cytoplasmic translocation initiates lipophagy; RNF186 ubiquitinates HMGB1 for degradation. The dashed lines indicate inhibitory paths.

Lipophagy maintains intracellular lipid equilibrium in MASLD. Studies on ring finger protein 186 (RNF186) knockout mice, human hepatocytes, and mouse primary hepatocytes explored RNF186's influence on lipophagy in MASLD.^24^ RNF186 depletion increased lipophagy in MASLD hepatocytes. RNF186 acts as an E3 ubiquitin ligase, ubiquitinating cytoplasmic high mobility group box 1 (HMGB1) at lysine 48 (K48) and 63 (K63) for proteasomal degradation. HMGB1 translocation from the nucleus to the cytoplasm initiates lipophagy in MASLD. HMGB1 knockdown decreased lipophagy activation and lipid buildup from RNF186 absence. Maintaining nuclear HMGB1 is vital for RNF186 function in MASLD. RNF186/HMGB1 levels in human MASLD correlate with lipophagy, suggesting similar impacts in human disease. RNF186 deficiency promotes hepatic lipophagy in MASLD by inhibiting HMGB1 ubiquitination/degradation.^24^ Targeting RNF186–HMGB1 interaction may prevent/manage MASLD^29^ (Fig. 2).

## Atherosclerosis

Atherosclerosis, a chronic inflammatory disease characterized by the accumulation of lipids and fibrous elements in large arteries, is a leading cause of heart attacks and strokes.^63^ Lipophagy plays a multifaceted role in atherosclerosis development, interacting with lipid metabolism, immune responses, and cellular homeostasis in the vascular wall. In atherosclerosis, lipophagy primarily regulates lipid accumulation and clearance in macrophages, key players in plaque formation.^16^ These immune cells internalize LDLs that penetrate the arterial wall, storing lipids in droplets. Efficient lipophagy clears these LDs, preventing macrophages from transforming into foam cells—a lipid-laden state hallmark of early atheroma.^64^

Disorders in lipid metabolism drive atherosclerosis progression. Studies on sirtuin 6 (Sirt6) highlight its role in enhancing macrophage autophagy and plaque stability. Using transmission electron microscopy and Bodipy 493/503 staining, researchers examined LDs in plaques from ApoE^−/−^ and ApoE^−/−^ Sirt6Tg mice. Findings showed that Sirt6 overexpression reduced necrotic cores and improved stability scores compared to controls.^65^ Reintroducing Sirt6 in models ameliorated lipid metabolism disorders and slowed atherosclerosis. Macrophages treated with acetylated LDL showed increased LDs and elevated PLIN2 levels, indicating lipid accumulation.^66^ Recombinant Sirt6, with SNF2H, suppressed Wnt1 expression, improving lipid disorders via lipophagy promotion. Conversely, Sirt6 knockdown in acetylated LDL-exposed macrophages impaired LD breakdown and promoted foam cells.^65^ This underscores the link of Sirt6 down-regulation to lipid metabolism disruption in atherosclerosis. Targeting Sirt6 for lipid regulation offers therapeutic potential for prevention and intervention.^67^

Macrophage autophagy is a vital anti-atherosclerotic mechanism facilitating LD breakdown and lipid homeostasis. Selective autophagy uses markers like ubiquitin and receptors (SARs) to target cargoes. “Lipophagy” originated from yeast studies on LDL degradation.^68^ Mass spectrometry analyzed LD proteomes in foam cells, revealing structural proteins like PLIN2, metabolic enzymes like acyl-CoA synthetase long-chain family member (ACSL), neutral lipases like patatin-like phospholipase domain containing 2 (PNPLA2), ubiquitination proteins like ancient ubiquitous protein 1 (AUP1), and autophagy-related proteins like HMGBs and tyrosine 3-monooxygenase/tryptophan 5-monooxygenase activation protein (YWHA/14-3-3).^69^ Functional significance of these 91 factors was assessed using siRNA arrays and cholesterol efflux assays. Knockdown of genes like Hmgb1, Hmgb2, Hspa5, and Scarb2 reduced efflux.^69^ SARs like SQSTM1/p62, neighbor of BRCA1 gene 1 (NBR1), and optineurin (OPTN) localized on LDs, suggesting phagocytic roles.^69^ Yeast lipophagy assays identified essential elements like HSPA5, UBE2G2, and AUP1 for lipophagy.^69^ This identification provides biological and therapeutic insights. Targeting these to enhance lipophagy and cholesterol efflux from foam cells could yield novel strategies for atherosclerosis.^70^

## Obesity

Obesity, characterized by excessive adipose tissue accumulation, is a major risk factor for metabolic diseases. Lipophagy, a selective form of autophagy targeting LDs, is crucial in obesity, where its dysfunction contributes to increased adiposity and compromised lipid mobilization.^21^ Microtubule-associated protein 1 light chain 3-II (LC3-II), a lipidated form of LC3 essential for autophagosome elongation and closure, interacts with p62/SQSTM1, an adapter protein that binds to ubiquitinated targets on LDs to facilitate degradation.^6^ However, in obesity, chronic mTOR pathway activation—a nutrient sensor suppressing autophagy—hinders these processes.^56^ Concurrently, AMPK, which promotes autophagy by inhibiting mTOR or activating ULK1, is down-regulated, reducing lipophagic activity and lipid clearance.^71^ This impairment exacerbates oxidative stress, as undegraded lipids overload mitochondria, generating ROS via electron transport chain leakage.^45^

Obesity is linked to metabolic disorders, posing a global health challenge. Understanding adipogenesis mechanisms is vital for treating excessive white fat accumulation. Y-box binding protein 1 (YBX1), a DNA- and RNA-binding protein, controls brown adipogenesis. YBX1 deficiency impairs adipocyte formation in mice and pigs, enhancing autophagy via ULK1 and ULK2. RNA immunoprecipitation shows YBX1 binds 5-methylcytosine (m5C)-modified Ulk1 mRNA, stabilizing it to boost ULK1 expression. YBX1 also transcriptionally up-regulates Ulk2, facilitating autophagy and adipogenesis. White adipose tissue YBX1 overexpression increases ULK1/ULK2-mediated autophagy, expanding adipose in mice.^72^ These findings highlight YBX1's control of autophagy/adipogenesis through post-transcriptional/transcriptional mechanisms, positioning YBX1 as a target for obesity therapies.^73^

**Figure 3:**
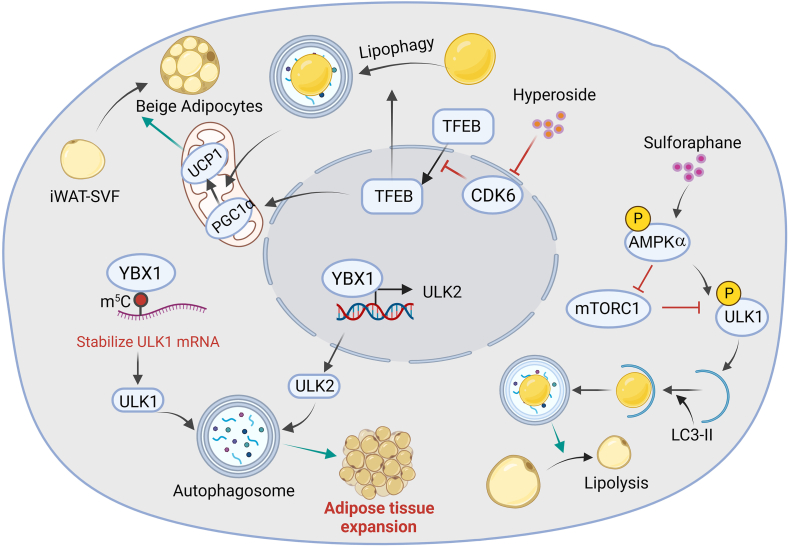
Role of lipophagy in obesity. i) Hyperoside effectively inhibits CDK6, which facilitates the increased nuclear translocation of TFEB. This translocation boosts the expression of UCP1 and enhances lipophagy, thereby increasing energy expenditure in white adipose tissue. This mechanism provides a protective strategy against obesity induced by a high-fat diet. ii) YBX1, recognized as a m5C-binding protein, selectively targets m5C-modified Ulk1 mRNA, enhancing ULK1 expression by stabilizing its mRNA. Furthermore, YBX1 serves as a transcription factor to augment Ulk2 transcription and expression, subsequently promoting both autophagy and adipogenesis. Sulforaphane activates AMPK phosphorylation, mTOR dephosphorylation, and ULK1 phosphorylation, leading to increased lipophagy. This enhanced lipophagy promotes partial lipolysis in adipocytes, contributing to the breakdown of fats.

Lipophagy interacts with hormonal/metabolic signaling in obesity. Insulin signaling, influencing mTOR via AKT serine/threonine kinase (AKT) phosphorylation, suppresses lipophagy under chronic hyperinsulinemia, common in early obesity.^74^ As insulin resistance emerges, erratic lipophagy contributes to adipose dysfunction and imbalance.^75^ Rubicon, negatively regulating autophagosome-lysosome fusion, and fat-specific protein 27 (FSP27), promoting LD growth, are elevated in obese adipose, stabilizing LDs and inhibiting degradation.^76^ This network's intricacy suggests targeting lipophagy therapeutically. Strategies include AMPK/mTOR modulators or gene therapies correcting autophagy-related gene expression.^56^ Such interventions restore lipophagic activity, reduce adiposity, and improve metabolic health.^20^ This underscores lipophagy's potential in obesity management (Fig. 3).

## Diabetes

Lipophagy plays a pivotal role in type 2 diabetes mellitus by preserving insulin sensitivity and regulating lipid metabolism, which are critical for managing hyperglycemia and preventing complications.^56^ This selective autophagic process eliminates excess LDs from cells, particularly in insulin-responsive tissues like the liver and skeletal muscle.^61^ Efficient lipophagy prevents the accumulation of harmful lipid intermediates that impair insulin signaling and exacerbate insulin resistance.^77^ However, in type 2 diabetes mellitus, lipophagy is frequently compromised, leading to metabolic disturbances that contribute to disease progression.^76^ This impairment amplifies oxidative stress, as undegraded lipids overload mitochondria, generating ROS via electron transport chain dysfunction and activating inflammatory pathways.^45^

Diabetes alters the expression and function of key ATG proteins. In diabetic models, reduced levels of ATG7 and LC3-II in liver and muscle tissues indicate autophagic dysfunction.^75^ These proteins are essential for autophagosome formation and elongation, enveloping LDs for degradation; their deficiency links to diminished fat removal and elevated cellular oxidative stress.^78^ Additionally, the adapter protein p62/SQSTM1, which targets LDs by bridging them to LC3 on autophagosomes, accumulates in diabetic tissues, signaling blocked autophagic flux.^76^ The interplay between lipophagy and insulin signaling is significant: efficient lipophagy clears lipid intermediates like diacylglycerol and ceramides, which activate protein kinase C (PKC) and inhibit insulin signaling.^79^ For instance, diacylglycerol/ceramide accumulation due to defective lipophagy activates PKC, impairing insulin receptor substrate-1 (IRS-1) phosphorylation and glucose uptake.^80^ Recent studies highlight this in diabetic kidney disease, where lipophagy impairment elevates ROS, disrupting glomerular function.

In summary, the relationship between lipophagy and type 2 diabetes mellitus involves multiple pathways and molecular players, with dysregulation fostering lipotoxicity and oxidative stress. Therapies targeting lipophagy enhancement could improve lipid turnover, reduce harmful intermediates, and boost insulin sensitivity. For example, docosahexaenoic acid up-regulates ATG7/12 to protect against palmitic acid-induced lipotoxicity in pancreatic β-cells.^81^ Trehalose induces autophagy to mitigate oxidative stress in Parkinson's models, with potential type 2 diabetes mellitus applications.^5^ By addressing these mechanisms, novel treatments may offer comprehensive type 2 diabetes mellitus management beyond symptom control.^56^

## Cancer

In cancer, the metabolic demands of rapidly proliferating cells necessitate significant alterations in energy production and nutrient management. Lipophagy, an important regulator of lipid processing, has a multifaceted impact on cancer growth, either supporting or hindering tumor development based on the type of cancer and its unique metabolic needs.^82^ Cancer cells often repurpose physiological processes to support their uncontrolled growth and survival, and lipophagy is no exception.^83^ By degrading LDs and releasing free fatty acids, lipophagy provides a vital source of energy that can fuel the high metabolic demands of tumor cells.^84^ This process is particularly crucial in environments where nutrient supplies are limited, such as within solid tumors, where rapidly proliferating cells may outpace the development of vascular supply.^85^

Periprostatic adipose tissue closely surrounds the prostate and is becoming more acknowledged for its involvement in the progression of prostate cancer.^85^ Cancer cells within this microenvironment can absorb lipids and sequester them in LDs, utilizing the released fatty acids as primary metabolic fuels.^86^ These fatty acids not only provide energy but also facilitate cancer progression. Recent research links these fatty acids to autophagy, a cellular recycling pathway, underscoring a complex interaction between autophagy, lipophagy, and cancer dynamics. In a comprehensive study, researchers examined the relationship between autophagy and lipophagy markers and aggressive characteristics in 465 human prostate cancer tissue samples. They examined different indicators, such as autophagy markers (p62, LC3), LD indicators (PLIN and oil red O), androgen receptor (AR) levels, proliferation marker (Ki67), and epithelial–mesenchymal transition indicator (Zeb1). *In vitro* evaluations were conducted in the study, involving the co-culturing of prostate cancer cell lines PC3 and 22RV1 with adipocytes from periprostatic adipose tissue of patients to investigate the impact of periprostatic adipose tissue on autophagic and lipophagic functions. The findings highlighted a significant correlation between LD markers and autophagy markers in prostate cancer tissues, which also correlated with clinical and biological indicators of disease aggressiveness. Particularly, PLIN staining was notably associated with AR expression. Elevated amounts of p62, LC3, and PLIN were detected in extraprostatic areas in instances of locally advanced prostate cancer, marking the locations where cancer cells directly interact with periprostatic adipose tissue. Furthermore, when prostate cancer cell lines were cultured with adipocytes, there was a reduction in autophagic activity and an increase in LD flux in PC3 cells, indicating a stimulated lipophagic response. These results underscore an active lipophagic process in prostate cancer that correlates with the aggressiveness of the disease, proximity to periprostatic adipose tissue, and responses observed under *in vitro* conditions involving adipocyte co-culture. These observations indicate that lipophagy is crucial in the advancement of prostate cancer, emphasizing possible areas for treatment targeting the control of lipid metabolism in the tumor microenvironment.^87^ This underscores the potential of targeting lipophagic pathways as a novel strategy for managing and possibly curbing prostate cancer progression.^88^

In hepatocellular carcinoma, cancer cells harness lipophagy to sustain energy production and support their rapid growth.^89^ Enhanced lipophagy in hepatocellular carcinoma cells facilitates the breakdown of LDs, releasing fatty acids that are subsequently β-oxidized to produce ATP. This mechanism is crucial for tumor survival and growth, particularly under hypoxic conditions where glycolytic pathways are less effective due to limited oxygen supply.^90^ Conversely, lipophagy can also suppress tumor development in certain contexts by maintaining cellular homeostasis and preventing the accumulation of lipotoxic intermediates that could induce cell death. In cancers like prostate cancer, where lipid accumulation is linked with tumor progression, actively maintaining lipophagy may help keep lipotoxicity at bay, potentially slowing the progression of the disease.^91^

## Neurodegenerative diseases

Neurodegenerative disorders, such as Alzheimer's disease, Parkinson's disease, and Huntington's disease, are characterized by progressive neuronal loss and dysfunction.^92^ Numerous studies indicate that abnormalities in lipid metabolism and autophagy pathways, including lipophagy, play essential roles in their pathogenesis.^93^ Lipophagy regulates LD turnover, influencing neuronal health, function, and neurotoxic protein aggregation—a hallmark of these conditions.^94^ In neurodegenerative contexts, neurons depend on coordinated lipophagy to manage lipid metabolism and mitigate lipotoxicity, which otherwise contributes to cell death.^95^ Neurons, highly reliant on efficient energy production and signaling, are vulnerable to lipid disruptions. Lipophagy maintains neuronal function by controlling LD levels, which store fatty acids for energy and membrane formation.^96^ However, disrupted lipophagy leads to abnormal LD accumulation, altering membrane composition, impairing signaling, and amplifying oxidative stress through mitochondrial overload and ROS generation.^97^

Parkinson's disease, the second most common neurodegenerative disorder after Alzheimer's disease, involves progressive loss of dopaminergic neurons in the ventral midbrain.^97^ This degeneration includes oxidative stress, mitochondrial dysfunction, proteostasis loss, and impaired autophagy.^98^ Recent evidence links lipid metabolism alterations to Parkinson's disease etiology, suggesting modulation as a therapeutic strategy.^99^ Researchers examined linoleic acid's neuroprotective and anti-inflammatory effects in Parkinson's disease models, using SH-SY5Y cells exposed to 6-hydroxydopamine (6-OHDA) and mice. *In vitro*, linoleic acid increased LD formation and improved autophagy/lipophagy flux, yielding antioxidant benefits.^5^ These findings position linoleic acid as a protective agent against Parkinson's disease, mitigating neurodegeneration in models. Additionally, mutations in leucine-rich repeat kinase 2 (LRRK2) affect autophagy, leading to dysfunctional mitochondria accumulation and heightened oxidative stress, impairing lipophagy and contributing to dopaminergic neuron loss.^96^ The LRRK2–lipophagy link emphasizes targeting lipid metabolism and autophagy for comprehensive Parkinson's disease management, potentially slowing progression.^96^

Neuroinflammation and neural damage, driven by microglial activation, are critical in diabetes-associated cognitive impairment.^98^ Microglial lipophagy, often overlooked, regulates lipid homeostasis and inflammation. Under high-glucose conditions, inhibited lipophagy causes LD accumulation in microglia.^98^ This was demonstrated in leptin receptor-deficient (db/db) mice, HFD/streptozotocin-induced type 2 diabetes mellitus mice, mouse microglia, and human HMC3 cells. Mechanistically, accumulated LDs interact with triggering receptor expressed on myeloid cells 1 (TREM1), an inflammation amplifier in microglia. This enhances TREM1 accumulation, worsening high-glucose-induced lipophagic damage and amplifying neuroinflammation via NLRP3 inflammasome. This cascade exacerbates diabetes-associated cognitive impairment. Pharmacological TREM1 inhibition with LP17 in db/db and HFD/streptozotocin mice reduced LD/TREM1 buildup.^98^ These decreases lowered hippocampal inflammation, improving cognition.^98^ The discovery reveals a novel mechanism where impaired lipophagy elevates TREM1, driving microglial activation and neuroinflammation in diabetes-associated cognitive impairment.^98^ Targeting the lipophagy–TREM1 axis could slow/reverse diabetic cognitive decline.^98^

Foamy macrophages, laden with residual myelin, are a key feature of multiple sclerosis.^100^ Reducing intracellular lipid load in these macrophages to promote a pro-repair phagocytic phenotype is a promising therapeutic strategy.^101^ Prolonged myelin-derived lipid storage biases phagocytes toward a disease-promoting, inflammatory phenotype. Autophagy, particularly LD-targeted lipophagy, is crucial. Autophagy disruption is a primary driver of this harmful phenotype.^101^ Studies investigated trehalose, a natural disaccharide, to induce autophagy, reducing lipid burden and inflammatory state in myelin-laden macrophages.^101^ Trehalose decreased lipid load, ameliorated inflammation, and facilitated myelin reformation in *ex vivo* brain slices and *in vivo* cuprizone demyelination models.^101^ These findings elucidate molecular processes underlying disease progression from impaired myelin-lipid degradation in macrophages. They highlight inducing phagocyte lipid metabolism as an effective strategy for myelin repair.^101^ This could advance treatments for multiple sclerosis and demyelinating diseases.^101^

Therapies enhancing lipophagy show promise in neurodegeneration. Linoleic acid induces lipophagy to counter oxidative stress in Parkinson's disease models.^5^ Trehalose restores flux in multiple sclerosis, reducing inflammation. LP17 targets TREM1 to alleviate lipophagy impairment in diabetes-associated cognitive impairment.^98^ Challenges include tissue-specific delivery; future directions emphasize AMPK/mTOR modulators for balanced autophagy.^56^

## Renal lipotoxicity

Renal lipotoxicity refers to the cellular damage and inflammation caused by excessive lipid accumulation in kidney tissues, often exacerbated by HFD in the context of metabolic diseases.^53^^,^^102^ Lysosomal dysfunction and impaired autophagic flux play central roles in this process. In mice, HFD leads to phospholipid accumulation in enlarged lysosomes within proximal tubular cells.^103^ Isolated renal tubular cells treated with palmitic acid exhibit progressive autophagic degradation decline, lysosomal acidification impairment, and excessive lipid buildup.^103^ Pulse-chase experiments confirm these lipids originate from cell membranes. In proximal tubular cell-specific autophagy-ablated mice, HFD-exposed proximal tubular cells show greater ubiquitin-positive protein aggregates—normally cleared by autophagy—than normal-diet controls.^103^ Autophagy ablation intensifies HFD effects on hyperuricemia-induced mitochondrial dysfunction and NLRP3 inflammasome activation.^104^ In HFD mice, renal ischemia-reperfusion injury is more severe than in normal-diet counterparts, worsened by the absence of autophagy.^103^ Human analyses reveal similar patterns: phospholipid accumulation in enlarged lysosomes and hindered autophagic flux in obese patients' kidneys versus nonobese.^103^ These findings highlight disrupted lysosomal/autophagic functions in renal lipotoxicity, underscoring potential targets for obesity-related kidney disease.^105^ This understanding of renal lipotoxicity pathophysiology suggests innovative treatments for obesity-associated chronic kidney disease.^102^

Lipophagy and mitophagy, selective autophagic processes targeting LDs and mitochondria, are essential for renal homeostasis and preventing diseases like acute kidney injury, chronic kidney disease, and diabetic nephropathy.^102^ In renal pathophysiology, lipophagy clears excess lipids to mitigate lipotoxicity, while mitophagy removes damaged mitochondria to reduce oxidative stress from ROS.^102^ Dysregulation in these pathways contributes to metabolic disorders, amplifying inflammation and tissue damage.^102^ Targeting lipophagy/mitophagy offers therapeutic strategies for renal disorders.^102^ Although primarily focused on male reproductive disorders, lipophagy's role in lipid homeostasis may extend to renal contexts, where disrupted lipid metabolism links to oxidative stress and infertility in metabolic syndromes.^53^

## Therapeutic implications of modulating lipophagy

Modulating lipophagy holds significant therapeutic potential across metabolic disorders like diabetes and obesity, neurodegenerative diseases, and cardiovascular conditions such as atherosclerosis.^45^ The ability to enhance or inhibit lipophagy pharmacologically offers avenues to correct lipid dysregulation, mitigate oxidative stress, and address pathologies.^54^ This section synthesizes specific modulators with demonstrated lipophagy specificity, verified mechanisms, and links to oxidative stress reduction.

## Hyperoside

Hyperoside (quercetin-3-O-β-d-galactopyranoside), a flavonoid glycoside from Hypericum perforatum (St. John's Wort) and Crataegus spp. (hawthorn), exhibits anti-inflammatory and antioxidant effects.^43^ In studies, hyperoside (80 mg/kg/day gavage) was administered to 4-week-old male C57BL/6J mice on a normal chow diet or HFD for 8 weeks, with 0.5% methylcellulose as vehicle.^106^ *In vitro*, it was tested on 3T3-L1 preadipocytes and primary stromal vascular fraction cells from mouse inguinal white adipose tissue.^106^ Hyperoside promoted white-to-beige fat conversion *in vivo* via uncoupling protein 1 (UCP1), improving glucose/lipid metabolism and protecting against HFD-obesity.^106^ It triggered lipophagy, a selective autophagy targeting LDs; autophagy inhibition reduced UCP1 expression, linking autophagic activity to browning.^106^ Mechanistically, hyperoside inhibited cyclin-dependent kinase 6 (CDK6), enhancing TFEB nuclear translocation and UCP1 expression.^106^ CDK6 overexpression partially reversed the benefits. These effects augment energy expenditure in white adipose tissue via CDK6–TFEB, alleviating oxidative stress from lipid overload.^107^ Hyperoside's pathway underscores its potential as an anti-obesity agent with broader metabolic implications.

## Leflunomide

Leflunomide, a prototype inhibitor of dihydroorotate dehydrogenase (DHODH)—a mitochondrial enzyme catalyzing dihydroorotate to orotate in *de novo* pyrimidine synthesis^108^—is used for rheumatoid arthritis.^109^ Rheumatoid arthritis patients on leflunomide experience weight loss and blood glucose reduction, beyond side effects.^109^ Its active metabolite, A77 1726, inhibits ribosomal protein S6 kinase 1 (S6K1), enhancing insulin receptor sensitivity and controlling hyperglycemia.^110^ In 3T3-L1 adipocytes, A77 1726 enhanced LC3 lipidation and autophagosome/lipolysosome formation via transforming growth factor-β-activated kinase 1 (TAK1), AMPK, and ULK1 activation, reducing LD accumulation.^111^ Bafilomycin A1 (autophagy inhibitor) or beclin-1 knockdown blocked LD reduction, confirming autophagy's role. Similar effects occurred in S6K1-deficient mouse embryonic fibroblast-derived adipocytes.^111^ In ob/ob mice, leflunomide curbed weight gain, reduced visceral fat and adipocyte size, and induced autophagy in adipose/liver tissues, lowering liver lipids. S6K1 knockout mice showed increased LC3 lipidation in liver, muscle, and fat; leflunomide/S6K1 deficiency phosphorylated TAK1, AMPK, and ULK1.^111^ These results highlight leflunomide's obesity regulation via AMPK activation and lipophagy promotion, suggesting repurposing for metabolic disorders.^111^ It mitigates oxidative stress from lipotoxicity.^108^

## Sulforaphane

Sulforaphane, a chemotherapeutic isothiocyanate, triggers lipolysis via hormone-sensitive lipase activation and white adipocyte browning.^112^ Recent studies examined sulforaphane's effects on autophagy in epididymal fat of HFD or normal diet-fed mice, and pathways in mature 3T3-L1 adipocytes.^20^ Western blotting showed sulforaphane increased autophagy-related LC3-II in 3T3-L1 adipocytes and HFD-fed mouse epididymal adipose.^20^ Immunofluorescence revealed LC3 colocalization with LDs in 3T3-L1 and perilipin in HFD-fed adipocytes.^20^ mCherry–EGFP–LC3 and GFP–LC3–RFP–LC3ΔG probes confirmed autophagic flux activation in 3T3-L1. Mechanism exploration via Western blotting showed ATG5 knockdown partially blocked sulforaphane-induced fatty acid release from LDs.^20^ In differentiated cells, sulforaphane induced AMPK phosphorylation, mTOR dephosphorylation, and ULK1 phosphorylation time-dependently. Thus, sulforaphane stimulates lipophagy via AMPK–mTOR–ULK1 signaling, contributing to partial lipolysis.^20^ This suggests that sulforaphane regulates lipid metabolism, beneficial for abnormal lipid accumulation disorders, reducing oxidative stress from lipotoxicity.^20^

## Geniposide

Geniposide, an iridoid glycoside from Gardenia jasminoides Ellis, improves atherosclerosis by regulating autophagy.^113^ Administered to apolipoprotein E-deficient (ApoE^−/−^) mice on HFD and oxidized LDL-treated primary vascular smooth muscle cells, geniposide reduced arterial lipid buildup, plaque progression, and collagen loss. Foam cell formation was assessed by lipid accumulation, inflammation, apoptosis, and markers. Network pharmacology explored geniposide's lipophagy in atherosclerosis. It enhanced lysosomal function, lipophagy markers, and lipid-marker colocalization. Chloroquine (lipophagy blocker) reversed geniposide's efficacy. Poly (ADP-ribose) polymerase 1 (PARP1) was investigated with olaparib (PARP1 inhibitor) and PARP1 overexpression. Geniposide reversed HFD-induced hyperlipidemia, plaque, and inflammation *in vivo*. *In vitro*, it suppressed foam cell formation of vascular smooth muscle cells by reducing lipid accumulation, apoptosis, and markers.^113^ Geniposide alleviated atherosclerosis by enhancing lipophagy via suppressing PARP1/PI3K/AKT signaling. PARP1 inhibition with olaparib enhanced lipophagy and alleviated progression, while overexpression increased foam cells and hindered lipophagy. PI3K inhibitor LY294002 moderated PARP1 effects, indicating that PARP1 disrupts ATP-binding cassette subfamily G member 1 (ABCG1)–PLIN1 interaction.^113^ Geniposide restores failed lipophagy via PARP1/PI3K/AKT, first revealing geniposide's lipophagy-promoting effects and PARP1's inhibitory role in ABCG1–PLIN1 crosstalk, providing perspectives for atherosclerosis treatments.^113^ It mitigates oxidative stress from lipotoxicity.

## Bisdemethoxycurcumin

Vascular smooth muscle cells contribute significantly to atherosclerotic foam cell formation.^114^ *In vitro*, bisdemethoxycurcumin (BDMC), with anti-inflammatory/antioxidant properties, was examined on oxidized LDL-stimulated vascular smooth muscle cells. BDMC reduced LD accumulation in oxidized LDL-stimulated vascular smooth muscle cells. It promoted autophagy by suppressing phosphoinositide-dependent protein kinase 1 (PDK1)/AKT/mTOR signaling.^114^ This pathway regulates cellular growth/survival; BDMC's inhibition shifts to the breakdown/removal of harmful lipids. *In vivo*, BDMC alleviated inflammation and lipid buildup in ApoE^−/−^ mice on HFD. BDMC shows promise for atherosclerosis prevention/treatment.^114^ By decreasing lipid accumulation and enhancing autophagy in vascular smooth muscle cells, BDMC addresses key atherosclerosis processes. It reduces oxidative stress from oxidized LDL.^115^

## Conclusion and perspective

This review has comprehensively explored lipophagy's critical roles in maintaining lipid homeostasis, regulating energy metabolism, and influencing disease pathogenesis, with a unique emphasis on its interplay with oxidative stress as a central link in metabolic disorders.^21^^,^^43^ As a specialized form of autophagy dedicated to degrading LDs, lipophagy supports cellular adaptation to energy demands and prevents pathological lipid accumulation, leading to lipotoxicity.^22^ Its interactions with metabolic pathways, particularly in counteracting ROS from mitochondrial overload and endoplasmic reticulum stress, underscore its importance in health and disease.^45^ Impaired lipophagy contributes to disorders like obesity, diabetes, MASLD, atherosclerosis, cancer, neurodegenerative conditions, and renal lipotoxicity, where it amplifies oxidative damage, inflammation, and tissue injury.^2^

Lipophagy modulation presents promising therapeutic opportunities. Enhancing it reduces lipid overload in metabolic syndromes, while selective inhibition may curb tumor growth in cancers exploiting the pathway.^83^ Pharmacological agents targeting regulators like AMPK and mTOR show efficacy in models; for example, fenofibrate promotes TFEB-mediated lipophagy to alleviate hepatic fat,^24^ and sulforaphane activates AMPK–mTOR–ULK1 for anti-obesity effects.^20^ Natural compounds like hyperoside (via CDK6–TFEB) and geniposide (via PARP1/PI3K/AKT suppression) restore lipophagy in obesity and atherosclerosis.^107^^,^^113^ These interventions not only correct lipid dysregulation but also mitigate oxidative stress, offering root-cause treatments.^45^

Despite the promise, challenges persist in clinical translation. Lipophagy mechanisms remain incompletely understood, especially tissue-specific interactions and ROS feedback loops impairing autophagy components. Potential off-target effects, such as excessive free fatty acid release exacerbating mitochondrial ROS, require careful consideration.^45^ Developing precise modulators to avoid unintended consequences in essential processes is crucial.

Future research should elucidate detailed regulatory networks in diverse contexts using advanced imaging and molecular tools for live-cell visualization. Sophisticated disease models mimicking human pathologies will evaluate efficacy and safety. Interdisciplinary approaches integrating biochemistry, pharmacology, and clinical medicine are essential for translating findings into treatments. Targeting lipophagy could revolutionize the management of lipid-related diseases, slowing progression and improving outcomes through balanced, tissue-specific interventions.

## CRediT authorship contribution statement

**Qingqing Zhao:** Investigation, Data curation. **Fei Qu:** Data curation. **Yi Jin:** Writing – review & editing, Writing – original draft, Supervision, Funding acquisition.

## Funding

This work was supported by the 10.13039/501100001809National Natural Science Foundation of China (No. 82401009), 10.13039/501100007129Shandong Provincial Natural Science Foundation (China) (No. ZR2025MS1456), and Research Start-up Fee for Introducing Talents to Jinan Central Hospital (China) (No. YJRC2023001).

## Conflict of interests

The authors declared no conflict of interests.
